# Early Stages of the Natriuretic Hormone Story

**DOI:** 10.3389/fendo.2014.00180

**Published:** 2014-11-11

**Authors:** Branislav Lichardus

**Affiliations:** ^1^School of Management, City University of Seattle Programs, Bratislava, Slovakia; ^2^Formerly affiliated with The Institute of Experimental Endocrinology, Slovak Academy of Sciences, Bratislava, Slovakia

**Keywords:** natriuretic hormone, cross-circulation experiments, Na,K-ATPase, anterior-third ventricle region-AV3V, cerebrospinal fluid sodium concentration

## Abstract

The paper reviews the early stages of the research on natriuretic hormone. The described experimental work was designed and accomplished in several internationally recognized laboratories where the author was invited to extend his projects. The cross-circulation experiments in animals with acutely increased extracellular fluid volume documented, that in the mechanism of natriuresis – besides a series of the physical natriuretic factors – there is still room for an active humoral natriuretic substance. This substance inhibited the sodium transporting enzyme, Na,K-ATPase, in the frog skin. Analogous inhibition of the renal Na,K-ATPase may be partly responsible for the increased sodium excretion. It was further shown that the extent of natriuresis is positively modulated by the concentration of sodium in the cerebrospinal fluid detected in the anterior-third ventricle region (AV3V) in the brain.

The first International “Symposium on Natriuretic Hormone” was held at the Smolenice Castle, the Congress Center of the Slovak Academy of Sciences in 1969, 12 years after the suggestion of Homer Smith that some such factor could exist (1) and 8 years since the first corroborative experimental data were presented by the team of Hugh de Wardener (2). At the next symposium organized by the same Institution, a decade later we dealt rather with “Natriuretic Hormones” (3). This review is a selected account on the elaboration of the early stages of the hypothesis on the existence of a natriuretic hormone in which I was privileged to participate.

## Cross-Circulation Experiments and “*rein au cou*”

In the experiments of de Wardener et al. (2), evidence was advanced for the transfer of a natriuretic material from the donor dog with expanded extracellular fluid volume (ECFV) by the infusion of saline to the cross-perfused recipient dog. The experiments were arranged in such a way that the provoked natriuresis and urine excretion in the recipient animal was neither a result of an increase of the glomerular filtration rate nor of the decrease of known circulating hormones, anti-natriuretic steroids, and vasopressin. Consequently, a third factor was suggested to be involved.

Our group, in pursuing this provocative idea, modified the cross-perfusion experiment in dogs. Only one kidney *in situ* in the recipient dog was cross-perfused under constant perfusion pressure by the donor’s blood in order to expose the recipient kidney to a larger concentration of a natriuretic material than in cross-perfusion of the whole animals. To prevent a possible natriuretic effect of blood dilution by saline infusion rather the blood volume of the donor dog was expanded by an “artificial blood” (suspension of homologous erythrocytes in 6% bovine albumin in Ringer–Locke solution). The cross-perfused kidney increased sodium and urine excretion following the blood volume expansion of the donor dog. However, this “transferred natriuresis” was less pronounced than natriuresis provoked by infusion of saline in the previous experiment of de Wardener. It was concluded that even if the natriuretic effect of blood dilution is eliminated the cross-perfusion experiments may reveal the appearance of a natriuretic factor in blood of the donor dog following its blood volume expansion (4). It was established in other experiments that the bovine albumin in the “artificial blood” *as such* was not critical for natriuresis evoked by blood volume expansion (5).

Yet, in another experimental set up, the homologous kidney was transplanted to the neck of a dog (“*rein au cou*”) in which vasopressin and creatinine were infused and DOCA was administered intramuscularly at least 3 h before the urine collection started. Subsequently, the blood volume of the dog with the transplanted kidney was expanded by infusion of homologous blood. The experimental conditions assured constant renal arterial and venous pressures in the transplanted kidney, a constant plasma oncotic pressure and constant hematocrit, glomerular filtration rate, and renal blood flow. Moderate, when compared with the renal output of the other kidney *in situ*, but significant increase in urine output and sodium excretion was observed by the transplanted kidney. Since non-hormonal and hormonal factors modulating sodium and water excretion were kept under control in the transplanted kidney, the results indicated more specifically than in previous experiments that a natriuretic humoral material might play a role in the mechanism of natriuresis provoked by blood volume expansion (6).

The operation of a blood-borne natriuretic factor in rat cross-circulation experiment was shown only when so-called sustained fluid volume expansion was achieved by urine reinfusion in the expanded donor animal. This procedure apparently intensified the natriuretic signal to the recipient animal (7).

## A Natriuretic or a Dilution of an Anti-Natriuretic Substance?

Our next attempt was to challenge the question that emerged from the cross-circulation experiments, namely, whether the appearance of a putative blood-borne natriuretic factor in animals with expanded ECFV was the result of an increased concentration of a natriuretic or a dilution of an anti-natriuretic substance. The experiments were performed on un-anesthetized cows, from which substantial volume of blood can be removed without reversing the effect induced by the expansion of their ECFV with 6% dextran in physiological saline (30 ml/kg b.w. at a rate of 100 ml/min). A blood sample of 1000 ml was withdrawn before the infusion and another one at the end of the infusion. The deproteinized plasma was applied intravenously to the assay rats in a volume of 0.2 ml. A non-significant tendency of control samples rather to decrease both the rate of urine flow and sodium excretion in the assay animals, was observed. On the other hand, the samples withdrawn during the ECFV expansion in cows produced statistically significant increases in urine flow, sodium excretion, and the tubular fractional sodium excretion in the assay rats. The sensitivity of the biological assay (i.e., the natriuretic activity applied in 0.2 ml of deproteinized plasma) might indicate that a separate low-molecular weight natriuretic factor might be involved, and that we are not dealing with a mere dilution effect of infusion on an anti-natriuretic activity (8).

The same plasma sample also decreased the short-circuit current representing active sodium transport in the frog skin by a transporting enzyme Na,K-ATPase (9, 10). Buckalew et al. (11) showed that an ultrafiltrate of blood from volume expanded dogs inhibited sodium transport in the toad bladder. It may be another indication for the presence of a natriuretic material in the tested plasma sample as it was proposed that the renal mechanism of natriuresis is via inhibition of the transporting enzyme in the nephron (12).

Further elaboration of the concept of an endogenous inhibitor of the Na,K-ATPase resulted in an attractive hypothesis linking the inhibitor to digoxin-like activity found in some organs, blood, and urine and to the pathogenesis of essential and low-renin arterial hypertension (13–16). Indeed, it was subsequently found that anti-digoxin serum decreased blood pressure in young rats with DOCA-salt hypertension (17). The same anti-digoxin serum, however, was ineffective in suppressing natriuresis induced by ECFV expansion with saline in rats (18). This finding illustrated at that time that the number and nature of substances represented by the endogenous digoxin-like activity had not been satisfactorily answered (19–21), which is true even now.

## Involvement of Peripheral and Central Nervous System in the Sodium Balance

The reflex regulation of renal water excretion originating in the stretch receptors of heart atria having the vagal nerves as the afferent limb and vasopressin as an efferent limb (Gauer–Henry reflex) was proposed to be applicable also to the mechanism of renal regulation of sodium excretion. The efferent limb of the reflex was presumed to be a natriuretic factor produced in the brain. However, we found that natriuresis induced by infusion of artificial blood in dogs with either innervated or denervated kidneys was not abolished by bilateral vagotomy. Thus, the analogy of reflex renal sodium control with a modified Gauer–Henry reflex did not seem to be primarily at play (22). We, unfortunately, did not offer at that time a more creative conclusion from these experiments. It is obvious today that – 14 years before the discovery of atrial natriuretic peptides – we missed a possibility to speculate about the role of *the heart as such* in the renal sodium excretion during the ECFV expansion. Our shortcoming in judgment was partly due to the fact that others found the afferent signal to travel along afferent sympathetic nerves and the spinal cord which, of course, were not interrupted by vagotomy.

It has been shown in various species of anesthetized or conscious experimental animals that increased concentration or dilution of sodium salts in the cerebrospinal fluid (CSF) interfere, respectively, with renal sodium excretion (23). In our studies on conscious sheep, the increase in the CSF sodium concentration and the simultaneous expansion of the ECFV resulted in a much higher increase in renal sodium excretion in comparison to the effect of ECFV expansion in animals with normal CSF sodium concentration. Dilution of CSF sodium prevented completely natriuresis following the ECFV expansion. This is thus another indication of that the brain may be involved in the control of renal regulation of ECFV by monitoring sodium concentration in CSF (24).

The periventricular organs that lack the blood–brain barrier seem to be critical for changes in CSF and also in the systemic ECF sodium concentration. A critical brain area where the sodium concentration or osmolality is monitored is probably the anterior wall of the third ventricle (AV3V). This conclusion is supported by experiments in conscious sheep with ablated AV3V. The non-lesioned control sheep were in a spontaneous water balance, whereas water balance in the animals with chronically ablated AV3V region was re-established by forced application of drinking water through intrarumenal tube. It was found that ablation of AV3V region blocks natriuresis to hypertonic but not isotonic NaCl load provided the lesioned sheep is in water balance. McKinley et al. (25, 26) suggested that the AV3V region has a role in regulation of renal Na excretion in conditions where the plasma Na concentration increases. It was further proposed that increased renal Na excretion in response to hypernatremia is another cerebrally mediated osmoregulatory response. However, the AV3V region does not seem to be critical for the mechanism of natriuresis induced by ECFV expansion with isotonic saline (24, 26, 27).

The posterior hypothalamus was also found to be involved in the sodium and ECFV balance. Lesions in the posterior nucleus of the hypothalamus are followed by a renal salt wasting syndrome in rats, cats, and man. In both animal experiments and clinical cases, it was shown that this type of negative sodium balance could not be corrected by adrenal steroids. We tried to identify in rats whether the nuclei in the posterior hypothalamus react to the disturbed sodium balance. The rats were given only a 2% saline to drink for 10 days. A control group drank tap water. The hypothalami were then sectioned and the volume of 200 cell nuclei was determined in each animal of the following nuclei: posterior, ventromedialis, dorsomedialis, and arcuate. The distribution of cell nuclear volume size showed a statistically significant decrease only in the posterior hypothalamic nucleus, which might suggest that it could have been specifically influenced by increased sodium concentration in blood and/or in CSF. At the time of this experiment, changes of volume of cell nuclei in the nucleus posterior hypothalami were taken for an indication of their neuroendocrine activity in connection with salt loading (28, 29).

## Conclusion

Using modified cross-perfusion experiments, in which the blood was not diluted, a methodology was put forward to exclude the natriuretic effect of physical factors. It was confirmed that during blood volume expansion a natriuretic or dilution of anti-natriuretic material was revealed.

It was shown, in an attempt to isolate the presumed substance playing a role in natriuresis following the blood or the whole ECF volume expansion that it may be a substance with a natriuretic activity.

The isolated substance also decreased the short-circuit current in the frog skin, which is an indication for its potential to decrease the activity of the transporting enzyme Na,K-ATPase. This action of the natriuretic substance could be its contribution to the renal mechanism of natriuresis following ECFV expansion.

It was shown in conscious sheep that the brain is involved in the control of renal regulation of ECFV by monitoring sodium concentration in CSF. A critical brain area, as indicated by others, where the sodium concentration or osmolality is monitored is the anterior wall of the third ventricle (AV3V).

Indirect evidence was presented for a neuroendocrine activity of the nucleus posterior hypothalami related to the sodium balance in rats.

## Conflict of Interest Statement

The author declares that the research was conducted in the absence of any commercial or financial relationships that could be construed as a potential conflict of interest.
